# Supplementary material for: Comparison of two proxies for the preconception weight using data from a pre-pregnancy cohort in Benin: Weight measured in the first trimester of pregnancy vs estimated by Thomas’ formula
Source: PLoS One. 2024 Nov 4;19(11):e0312840. doi: 10.1371/journal.pone.0312840 (PMC11534216; doi:10.1371/journal.pone.0312840)
Supplement: S1 Table — (DOCX) [file pone.0312840.s001.docx]

**S1 Table:** **Sociodemographic and anthropometric characteristics of excluded (n=109) vs. included women (n=302). RECIPAL study in Benin, 2014-2017**

| Characteristics | Unit or Category | Mean ± SD or % (n=302) | Mean ± SD or % (n=109) | *p*-Value^¥^ |
| --- | --- | --- | --- | --- |
| Maternal height | cm | 157.9 ± 6.1 | 159.3 ± 5.0 | 0.03 |
| Maternal weight at inclusion for pre-pregnancy follow-up | kg | 56.9 ± 11.3 | 60.5 ± 11.1 | 0.001 |
| Pre-pregnancy BMI (at inclusion for pre-pregnancy follow-up) | kg/m^2^ | 22.8 ± 4.3 | 23.9 ± 4.4 | 0.03 |
|  | < 18.5 | 9.1 | 5.5 | 0.0001 |
|  | ≥ 18.5 and < 25 | 66.2 | 59.6 |  |
|  | ≥ 25 | 24.7 | 34.9 |  |
| Maternal age | Year | 26.7 ± 5.1 | 26.9 ± 4.7 | 0.79 |
| Area of residence | Sô-ava | 69.8 | 85.3 | 0.04 |
|  | Akassato | 30.2 | 14.7 |  |
| Ethnic group | Toffin | 69.2 | 83.5 | 0.02 |
|  | Aïzo | 16.2 | 9.2 |  |
|  | Others | 14.6 | 7.3 |  |
| Marital status | Unmarried cohabitation | 5.2 | 8.3 | 0.42 |
|  | Married monogamous | 65.5 | 66.0 |  |
|  | Married polygamist | 29.3 | 25.7 |  |
| Education level | Illiterate | 70.1 | 73.4 | 0.13 |
|  | Primary/literate | 18.8 | 22.0 |  |
|  | Middle or high school or Higher education | 11.1 | 4.6 |  |
| Women's professional status | Active | 93.5 | 97.3 | 0.3 |
|  | Unemployed | 4.9 | 0.9 |  |
|  | In training | 1.6 | 1.8 |  |
| Parity | 0 | 10.7 | 15.6 | 0.50 |
|  | 1 | 18.8 | 13.8 |  |
|  | 2 ≤ and <5 | 50.1 | 50.4 |  |
|  | ≥ 5 | 20.4 | 20.2 |  |
| ^¥^ T-test for interval variables, chi-square test for categorical variables | | | | |
