# Supplementary material for: Comparison of two proxies for the preconception weight using data from a pre-pregnancy cohort in Benin: Weight measured in the first trimester of pregnancy vs estimated by Thomas’ formula
Source: PLoS One. 2024 Nov 4;19(11):e0312840. doi: 10.1371/journal.pone.0312840 (PMC11534216; doi:10.1371/journal.pone.0312840)
Supplement: S2 Table — (DOCX) [file pone.0312840.s002.docx]

**S2 Table: Distribution of BMI categories according to the pre-pregnancy weight estimate used to calculate BMI (n=302). RECIPAL study, Benin, 2014-2017.**

| BMI (kg/m^2^) | BMI calculated using the reference pre-pregnancy weight and its two proxies | | | | | |
| --- | --- | --- | --- | --- | --- | --- |
|  | **MPPW** | | **FTPW** | | **EPPW** | |
|  | n | % | n | % | n | % |
| < 18.5 | 28 | 9.3 | 28 | 9.3 | 24 | 8.0 |
| ≥ 18.5 and <25 | 196 | 64.9 | 199 | 65.9 | 200 | 66.2 |
| ≥ 25 and < 30 | 54 | 17.9 | 52 | 17.2 | 52 | 17.2 |
| ≥ 30 | 24 | 7.9 | 23 | 7.6 | 26 | 8.6 |
| MPPW: Measured pre-pregnancy weight, FTPW: First Trimester of Pregnancy Weight, EPPW: Estimated Pre-pregnancy Weight using Thomas et al. formula. | | | | | | |
