# Supplementary material for: Comparison of two proxies for the preconception weight using data from a pre-pregnancy cohort in Benin: Weight measured in the first trimester of pregnancy vs estimated by Thomas’ formula
Source: PLoS One. 2024 Nov 4;19(11):e0312840. doi: 10.1371/journal.pone.0312840 (PMC11534216; doi:10.1371/journal.pone.0312840)
Supplement: S3 Table — (DOCX) [file pone.0312840.s003.docx]

**S3 Table: Description of the FTPW and the corresponding EPPW, according to the gestational age at measurement. RECIPAL study, Benin, 2014-2017**

| **Pre-pregnancy weight estimates** | **Gestational age ranges at the time of weight measurement** | **Descriptive statistical parameters of FTPW and EPPW** | | | | | | |
| --- | --- | --- | --- | --- | --- | --- | --- | --- |
|  |  | **Sample size** | **Mean ± SD** | **First quartile** | **Median** | **Third quartile** | **Minimum** | **Maximum** |
| **FTPW** | Age ≤ 5 | 98 | 56.53± 11.36 | 49.6 | 53.6 | 61.7 | 33.9 | 115.9 |
| **EPPW** | Age ≤ 5 | 98 | 57.29± 11.15 | 50.7 | 54.5 | 62.4 | 34.3 | 115.4 |
| **FTPW** | 5 < Age <7 | 81 | 59.37± 12.35 | 50.5 | 57.0 | 65.5 | 39.1 | 99.5 |
| **EPPW** | 5 < Age <7 | 81 | 59.95± 11.15 | 51.2 | 57.9 | 66.6 | 40.4 | 99.6 |
| **FTPW** | 7 ≤ Age < 9 | 99 | 56.37± 11.47 | 49.1 | 53.7 | 62.1 | 35.6 | 105 |
| **EPPW** | 7 ≤ Age < 9 | 99 | 56.91± 11.19 | 49.7 | 54.2 | 62.5 | 36.7 | 104.6 |
| **FTPW** | 9 ≤ Age < 11 | 100 | 55.54± 9.21 | 49.1 | 54.1 | 61.2 | 33.4 | 84.2 |
| **EPPW** | 9 ≤ Age < 11 | 100 | 55.96± 9.01 | 49.7 | 54.6 | 61.6 | 33.5 | 84.1 |
| **FTPW** | 11 ≤ Age < 13 | 118 | 56.44± 11.58 | 49.0 | 53.8 | 60.8 | 36.4 | 104.1 |
| **EPPW** | 11 ≤ Age < 13 | 118 | 56.70± 11.31 | 49.8 | 54.2 | 61.2 | 37.2 | 103.4 |
| **FTPW** | 13 ≤ Age < 14 | 65 | 56.72± 12.53 | 49.9 | 54.2 | 61.5 | 33.6 | 115.1 |
| **EPPW** | 13 ≤ Age < 14 | 65 | 56.80± 12.23 | 50.6 | 54.3 | 62.0 | 33.4 | 113.9 |
| FTPW: First Trimester of Pregnancy Weight, EPPW: Estimated Pre-pregnancy Weight using Thomas et al. formula. | | | | | | | | |
