# Supplementary material for: Comparison of two proxies for the preconception weight using data from a pre-pregnancy cohort in Benin: Weight measured in the first trimester of pregnancy vs estimated by Thomas’ formula
Source: PLoS One. 2024 Nov 4;19(11):e0312840. doi: 10.1371/journal.pone.0312840 (PMC11534216; doi:10.1371/journal.pone.0312840)
Supplement: S4 Table — (DOCX) [file pone.0312840.s004.docx]

**S4 Table:** **Raw value (kg) and percentage variation in the FTPW and the corresponding EPPW. RECIPAL study, Benin, 2014-2017**

| **Pre-pregnancy weight estimates** | **GA ranges (weeks)** | **Sample size** | | **^£^Amplitude of variation**  **(Mean ±SD) in kg and in %** | | | | **Concordance correlation coefficient (95% CI)** | |
| --- | --- | --- | --- | --- | --- | --- | --- | --- | --- |
|  |  | Within 3 months^¥^ | All participants | Within 3 months^¥^ | | All participants | | Within 3 months^¥^ | All participants |
|  |  |  |  | M ±SD in kg | M ±SD in % | M ±SD in kg | M ±SD in % |  |  |
| **FTPW** | ≤ 5 | 98 | 118 | - 0.21 ±1.61 | - 0.36 ±2.74 | - 0.07 ±2.01 | - 0.15 ±3.29 | 0.99 (0.98; 0.99) | 0.98 (0.98; 0.99) |
| **EPPW** | ≤ 5 | 98 | 118 | + 0.55 ±1.64 | +1.09 ±2.85 | + 0.66 ±2.05 | +1.22 ±3.38 | 0.98 (0.98; 0.99) | 0.98 (0.97, 0.98) |
| **FTPW** | 5 < Age <7 | 81 | 96 | - 0.13 ±1.99 | - 0.29 ±.3.42 | - 0.13 ±2.14 | - 0.27 ±3.63 | 0.98 (0.98; 0.99) | 0.98 (0.97, 0.99) |
| **EPPW** | 5 < Age <7 | 81 | 96 | + 0.44 ±1.95 | +0.80 ±3.49 | +0.43 ±2.13 | + 0.78 ±3.65 | 0.98 (0.98; 0.99) | 0.98 (0.97; 0.99) |
| **FTPW** | 7 ≤ Age < 9 | 99 | 136 | - 0.07 ±2.24 | - 0.19 ±.4.01 | - 0.13 ±2.17 | - 0.28 ±.3.86 | 0.98 (0.97; 0.98) | 0.98 (0.97; 0.98) |
| **EPPW** | 7 ≤ Age < 9 | 99 | 136 | + 0.46 ±2.27 | +0.89 ±4.04 | + 0.38 ±2.20 | +0.72 ±3.89 | 0.97 (0.97; 0.98) | 0.97 (0.97; 0.98) |
| **FTPW** | 9 ≤ Age < 11 | 100 | 121 | - 0.5 ±2.41 | - 0.79 ±.4.14 | - 0.37 ±2.43 | - 0.53 ±.4.17 | 0.96 (0.95; 0.97) | 0.97 (0.96; 0.98) |
| **EPPW** | 9 ≤ Age < 11 | 100 | 121 | + 0.08 ±2.40 | +0.05 ±4.16 | + 0.06 ±2.46 | +0.31 ±4.26 | 0.96 (0.95; 0.97) | 0.97 (0.96; 0.98) |
| **FTPW** | 11 ≤ Age < 13 | 118 | 156 | - 0.36 ±2.66 | -1.31 ±4.77 | - 0.21 ±2.72 | - 0.42 ±.4.77 | 0.97 (0.96; 0.98) | 0.97 (0.96; 0.98) |
| **EPPW** | 11 ≤ Age < 13 | 118 | 156 | + 0.10 ±2.64 | + 0.68 ±.4.80 | + 0.03 ±2.70 | +0.08 ±4.76 | 0.97 (0.96; 0.98) | 0.97 (0.96; 0.98) |
| **FTPW** | 13 ≤ Age < 14 | 65 | 115 | +0.06 ±2.71 | +0.24 ±.4.80 | +0.32 ±2.55 | +0.74±.4.61 | 0.97 (0.96; 0.98) | 0.97 (0.96; 0.98) |
| **EPPW** | 13 ≤ Age < 14 | 65 | 115 | + 0.14 ±2.67 | + 0.48 ±4.77 | + 0.36 ±2.53 | + 0.90 ±4.62 | 0.97 (0.96; 0.98) | 0.97 (0.96; 0.98) |
| ^£:^ To calculate the weight variation, the measured pre-pregnancy weight (MPPW) was subtracted from each FTPW and EPPW. So, any value preceded by the sign minus (-) indicates weight loss and sign plus (+) indicates weight gain.  ^¥:^ Group of study participants having the MPPW measured within 3 months before pregnancy start.  MPPW: Measured pre-pregnancy weight, FTPW: First Trimester of Pregnancy Weight, EPPW: Estimated Pre-pregnancy Weight using Thomas et al. formula. | | | | | | | | | |
