# Supplementary material for: Comparison of two proxies for the preconception weight using data from a pre-pregnancy cohort in Benin: Weight measured in the first trimester of pregnancy vs estimated by Thomas’ formula
Source: PLoS One. 2024 Nov 4;19(11):e0312840. doi: 10.1371/journal.pone.0312840 (PMC11534216; doi:10.1371/journal.pone.0312840)
Supplement: S5 Table — (DOCX) [file pone.0312840.s005.docx]

**S5 Table:** **Bland and Altman comparison parameters of the MPPW to the FTPW and to the corresponding EPPW, by gestational age ranges in the first trimester of pregnancy and according to the timing of measurement of the MPPW. RECIPAL study, Benin, 2014-2017**

| GA ranges (weeks) | Bland and Altman comparison parameters^£^ | Measurement of MPPW within 3 months before pregnancy | | All participants | |
| --- | --- | --- | --- | --- | --- |
|  |  | FTPW | EPPW | FTPW | EPPW |
| ≤ 5 | Range of differences (kg) | -5.6; +5.0 | -5.0; +5.5 | -7.5; 5.6 | -7.4; +6.4 |
|  | Mean difference (95% CI), in kg | +0.21(-0.11; +0.53) | -0.55(-0.88; -0.22) | +0.07(-0.30; +0.44) | -0.43(-1.04; -0.30) |
|  | Limits of agreement (kg) | -2.94; +3.37 | -3.77; +2.67 | -3.88; +4.02 | -4.68; +3.36 |
|  | 95% CI of lower limit of agreement (kg) | -3.56; - 2.45 | -4.40; - 3.27 | -4.58; - 3.32 | -5.40; - 4.11 |
|  | 95% CI of upper limit of agreement (kg) | +2.88; +3.99 | +2.17; +3.30 | +3.46; + 4.72 | +2.78; + 4.07 |
|  | Percentage of weights outside the limits of agreement (%) | 6.12 | 6.12 | 09.32 | 10.17 |
| 5 < Age <7 | Range of differences (kg) | -5.8; +4.2 | -5.0; +5.6 | -5.7; +5.6 | -5.7; +5.6 |
|  | Mean difference (95% CI), in kg | +0.13(-0.31; +0.57) | -0.44(-0.87; -0.01) | +0.13(-0.31; +0.56) | -0.43(-0.86; -0.00) |
|  | Limits of agreement (kg) | -3.77; +4.03 | -4.27; +3.39 | -4.06; +4.32 | -4.60; +3.73 |
|  | 95% CI of lower limit of agreement (kg) | -4.63; - 3.11 | -5.11; - 3.62 | -4.90; - 3.41 | -5.43; - 3.95 |
|  | 95% CI of upper limit of agreement (kg) | +3.38; +4.89 | +2.74; +4.23 | +3.66; + 5.16 | +3.08; + 4.57 |
|  | Percentage of weights outside the limits of agreement (%) | 6.17 | 7.41 | 7.29 | 6.25 |
| 7 ≤ Age < 9 | Range of differences (kg) | -5.5; +8.7 | -5.5; +8.7 | -6.0; +8.7 | -5.5; 8.7 |
|  | Mean difference (95% CI), in kg | +0.07(-0.38; +0.52) | -0.46(-0.91; -0.01) | +0.13(-0.23; +0.50) | -0.38(-0.75; -0.00) |
|  | Limits of agreement (kg) | -4.32; +4.46 | -4.91; +3.98 | -4.13; +4.39 | -4.68; +3.93 |
|  | 95% CI of lower limit of agreement (kg) | -5.18; - 3.64 | -5.53; + 4.61 | -4.82; - 3.56 | -5.39; - 4.11 |
|  | 95% CI of upper limit of agreement (kg) | +3.78; + 5.32 | +3.30; + 4.86 | +3.83; + 5.09 | +3.35; + 4.63 |
|  | Percentage of weights outside the limits of agreement (%) | 4.04 | 6.06 | 5.15 | 5.15 |
| 9 ≤ Age < 11 | Range of differences (kg) | -7.1; +7.1 | -6.7; +6.7 | -7.1; +7.1 | -6.7; 6.7 |
|  | Mean difference (95% CI), in kg | +0.50(+0.02; +0.98) | -0.08(-0.40; +0.55) | +0.37(-0.07; +0.81) | -0.06(-0.50; +0.38) |
|  | Limits of agreement (kg) | -4.22; +5.22 | -4.63; +4.78 | -4.39; +5.13 | -4.89; +4.77 |
|  | 95% CI of lower limit of agreement (kg) | -5.14; - 3.50 | -5.55; - 3.91 | -5.22; - 3.72 | -5.73; - 4.21 |
|  | 95% CI of upper limit of agreement (kg) | +4.50; + 6.14 | +4.06; + 5.70 | +4.46; + 5.97 | +4.09; + 5.61 |
|  | Percentage of weights outside the limits of agreement (%) | 7.00 | 5.00 | 6.61 | 4.96 |
| 11 ≤ Age < 13 | Range of differences (kg) | -6.9; +6.0 | -6.5; +6.1 | -6.9; +6.6 | -6.5; +6.4 |
|  | Mean difference (95% CI), in kg | +0.36(-0.13; +0.84) | -0.10(-0.38; +0.58) | +0.21(-0.22; 0.64) | -0.03(-0.45; 0.40) |
|  | Limits of agreement (kg) | -4.87; +5.58 | -5.07; +5.27 | -5.12; +5.54 | -5.33; +5.27 |
|  | 95% CI of lower limit of agreement (kg) | -5.79; - 4.12 | -5.99; - 4.34 | -5.93; - 4.45 | -6.13; - 4.66 |
|  | 95% CI of upper limit of agreement (kg) | +4.84; + 6.51 | +4.54; + 6.19 | +4.87; + 6.35 | +4.61; + 6.07 |
|  | Percentage of weights outside the limits of agreement (%) | 6.78 | 5.08 | 5.77 | 4.49 |
| 13 ≤ Age < 14 | Range of differences (kg) | -7.9; 9.4 | -7.7; +9.0 | -7.9: +9.4 | -7.7; +9.0 |
|  | Mean difference (95% CI), in kg | -0.06(-0.73; +0.61) | -0.14(-0.81; +0.52) | -0.32(-0.80; 0.15) | -0.36(-0.83; 0.11) |
|  | Limits of agreement (kg) | -5.37; +5.25 | -5.38; +5.09 | -5.33; +4.69 | -5.32; +4.60 |
|  | 95% CI of lower limit of agreement (kg) | -6.70; - 4.38 | -6.69; - 4.41 | -6.24; - 4.61 | -6.22; - 4.60 |
|  | 95% CI of upper limit of agreement (kg) | +4.26; + 6.58 | +4.12; + 6.41 | +3.96; + 5.59 | +3.88; + 5.49 |
|  | Percentage of weights outside the limits of agreement (%) | 4.62 | 4.62 | 4.39 | 4.39 |
| ^£:^ To calculate the weight variation using Bland and Altman method, each FTPW and EPPW was subtracted from the MPPW. So, any value preceded by the sign minus (-) indicates weight gain and sign plus (+) indicates weight loss  MPPW: Measured pre-pregnancy weight, FTPW: First Trimester of Pregnancy Weight, EPPW: Estimated Pre-pregnancy Weight using Thomas et al. formula. | | | | | |
