# Supplementary material for: Comparison of two proxies for the preconception weight using data from a pre-pregnancy cohort in Benin: Weight measured in the first trimester of pregnancy vs estimated by Thomas’ formula
Source: PLoS One. 2024 Nov 4;19(11):e0312840. doi: 10.1371/journal.pone.0312840 (PMC11534216; doi:10.1371/journal.pone.0312840)
Supplement: Attachment — Submitted filename: Response to reviewers.docx [file pone.0312840.s007.docx]

**Dear editor,**

Thank you for considering our manuscript PONE-D-24-21305, entitled “Comparison of two proxies for the preconception weight using data from a pre-pregnancy cohort in Benin: weight measured in the first trimester of pregnancy vs estimated by Thomas’ formula”.

We have revised the issues brought up by the reviewers and a point-by-point response can be found below. Please note that, as part of the process of taking comments into account, we have added two tables to the supporting information files.

We agree with the amended statement about the role of the funders that you suggested: "The funders had no role in study design, data collection and analysis, decision to publish, or preparation of the manuscript. But the IRD who funded the PhD grant."

Finally, as requested, we are submitting a clean and a track changes versions of the manuscript. Please note that in the present rebuttal letter all line numbers indicating where changes have been made in the manuscript refer to the version with track changes.

With kind regards,

Dr Emmanuel Koffi YOVO

Clinical Research Institute of Benin (IRCB)

04 BP 1114, Abomey-Calavi

Email: emkoffiyovo@gmail.com

Tel: +229 97 42 08 01

**Response to the academic editor**

Journal Requirements:

1. When submitting your revision, we need you to address these additional requirements.

Please ensure that your manuscript meets PLOS ONE's style requirements, including those for file naming. The PLOS ONE style templates can be found at

<https://journals.plos.org/plosone/s/file?id=wjVg/PLOSOne_formatting_sample_main_body.pdf>

The format of chapter headings, tables and file names has been revised to conform to the journal's requirements.

<https://journals.plos.org/plosone/s/file?id=ba62/PLOSOne_formatting_sample_title_authors_affiliations.pdf>

The formatting of authors affiliations has been checked and modified when necessary to comply with the journal’s requirements.

2. Please include a complete copy of PLOS’ questionnaire on inclusivity in global research in your revised manuscript. Our policy for research in this area aims to improve transparency in the reporting of research performed outside of researchers’ own country or community. The policy applies to researchers who have travelled to a different country to conduct research, research with Indigenous populations or their lands, and research on cultural artefacts. The questionnaire can also be requested at the journal’s discretion for any other submissions, even if these conditions are not met. Please find more information on the policy and a link to download a blank copy of the questionnaire here: <https://journals.plos.org/plosone/s/best-practices-in-research-reporting>. Please upload a completed version of your questionnaire as Supporting Information when you resubmit your manuscript.

The questionnaire has been completed and submitted as supporting information as requested.

3. Thank you for stating the following financial disclosure: "This work was supported by the French Agence Nationale de la Recherche (grant number ANR-13-JSV1-0004) and the Foundation Simone Beer under the auspices of the Fondation de France (grant number 00074147). EY received ARTS (Allocations de Recherche pour une Thèse au Sud), the original French name of a PhD grants programme from the French National Research Institute for Sustainable Development (IRD) for doing his PhD at Montpellier University".

Please state what role the funders took in the study.  If the funders had no role, please state: "The funders had no role in study design, data collection and analysis, decision to publish, or preparation of the manuscript. But the IRD who funded the PhD grant "

If this statement is not correct you must amend it as needed.

Please include this amended Role of Funder statement in your cover letter; we will change the online submission form on your behalf.

We agree with the above suggested statement, and we have included it in our cover letter as requested.

4. We note that you have included the phrase “data not shown” in your manuscript. Unfortunately, this does not meet our data sharing requirements. PLOS does not permit references to inaccessible data. We require that authors provide all relevant data within the paper, Supporting Information files, or in an acceptable, public repository. Please add a citation to support this phrase or upload the data that corresponds with these findings to a stable repository (such as Figshare or Dryad) and provide and URLs, DOIs, or accession numbers that may be used to access these data. Or, if the data are not a core part of the research being presented in your study, we ask that you remove the phrase that refers to these data.

Thanks, the phrase has been reformulated for more clarity, by referring to Figure 2 which contains all the necessary data (lines 383 to 387, in the revised version with track changes)

**Additional Editor Comments:**

The authors present an evaluation of two methods to determine pre-pregnancy weight, first trimester pregnancy weight and the use of Thomas’ formula, comparing agreement between the methods and between each method and true pre-pregnancy weight in a cohort of women from Benin. First trimester weights slightly underestimated pre-pregnancy weight (by mean ~150g) while the application of Thomas’ formula overestimated pre-pregnancy weight by a small bit greater degree (mean 430g). ~6% of weights were outside of the limits of the agreement between the method and the measured pre-pregnancy weight. Having a measured pre-pregnancy weight is a strength as is applying the analyses in sub-Saharan Africa.

The manuscript is well-written and clear. The variables are clearly defined.

1. As noted by reviewer 2, please could you clarify how gestational weight gain was determined?

Thanks for the request. Please see below my responses to reviewer 2 comment regarding this point.

2. Some of the language could be clarified. E.g., ‘concubinage’ is not a conventional term. Do you mean unmarried?

Thanks for the suggestion. Concubinage has been replaced by "Unmarried cohabitation” in Table1 (page 14 in the manuscript version with track changes).

3. The limitations of the study could be discussed in more detail.

The limitations are now more discussed, particularly after considering the reviewer 2 comments. More specifically, two points were added: Firstly, about the unavailability of data on self-reported pre-pregnancy weight, we specified that this was because we present here a secondary analysis of the RECIPAL study, which didn’t plan to analyze such kind of data. Secondly, about the representativeness of the women considered in the analyses, versus those who were excluded, we now present the results of the comparison between these groups in a new table (S1 Table). Please see also our reply to comment #6 of reviewer #2 below and lines 460 to 472.

Row 195: wg – does this indicate week?

Yes, wg stands for weeks of gestation. Please note that this abbreviation was previously defined (lines 155-156).

199. 201: language is not scientific. You have the numbers so no need for, ‘around’. In addition, use of number (2) and words (three). How numbers are expressed should be consistent and aligned with PLoSone guidelines.

The word “around” has been deleted (line 285 and 288) and numbers expression has been corrected to align with the journal’s guidelines (lines 285 and 289).

Table 1 – will need to be formatted in line with PLoS guidelines.

Table1 formatting has been corrected to align with the journal’s guidelines.

Parity. What is Unit?

Parity is the number of deliveries the mother got after 20 weeks of pregnancy before her inclusion in the study.

Is measure pre-pregnancy weight at 3 months pre-pregnancy?

Not exactly; indeed, the pre-pregnancy weight was measured *within* 3 months’ time before pregnancy start. We understand that this comment is linked to the lack of clarity at the end of the statistical paragraph. All this paragraph has now been reworded as requested by reviewer 2 (lines 175 to 216).

**Response to Reviewer # 1**

This is a well-written and interesting study. The authors provide convincing rationale for why to examine this question in a lower income, sub-Saharan country (this question has been examined in higher-income countries). The study upon which these secondary analyses were based was well-designed, with frequent measured pre-pregnancy weights.

1) Line 110: I believe the authors mean "its" rather than "it's"

Thank you very much for the kind words and for accepting to review our paper. This typo has been corrected (line 134, in the version with track changes of the manuscript).

2) The authors appear to have missed one previous study that has investigated this question, and it would be important to compare and contrast the current findings with this previous study.

Krukowski, R. A., West, D. S., DiCarlo, M., Shankar, K., Cleves, M. A., Saylors, M. E., & Andres, A. (2016). Are early first trimester weights valid proxies for preconception weight? BMC pregnancy and childbirth, 16, 1-6.

Thanks for bringing this article to our attention.

**Response to Reviewer # 2**

I appreciate the opportunity to review this paper. The problem of the mother’s weight at the beginning of pregnancy is important, especially in weight-gain studies, and has been understudied.

This study has one major positive point which is a measured pre-pregnancy weight (within 3 months prior to conception) to be used as a gold standard. However, two major points are worth attention.

1. The authors used gestational weight gain (GWG) to identify the gestational age at which weight gain starts to become significant, but they did not explain how GWG was calculated. This poses a substantial problem because the point will change if you use one value (pre-pregnancy weight) or the other (first-trimester weight) to calculate GWG.

Thank you for your positive comment. The gestational weight gains were calculated by subtracting the measured pre-pregnancy weight from each weight measurement during pregnancy. This is now clarified (lines 158-160, in the version with track changes of the manuscript).

2. The major issue is that it is not clear why not deriving an equation in the study to estimate the weight at conception based on first-trimester weight. Applying an equation developed for a very different North American population (and in a low sample) in this study is not well justified, especially because the authors had a ‘gold standard’ (the weight measured before the conception). Why not derive the ‘error’ from the first-trimester weight from the measured pre-pregnancy weight (the ‘gold standard’) in those individuals who had both and use this equation to predict the pre-pregnancy weight for those with first-trimester weight only? Considering that the authors concluded that the equation does not represent any gain for this population (it might not apply to them - as expected!), it is unclear to me the rationale for doing this analysis in the first place.

Thanks for the suggestion. Methodologically, your suggestion is interesting but from a different perspective. This approach would indeed allow us to have an equation specifically adapted to the context of our study. However, the chosen option for the analyses in this study was primarily to assess the performance of two proxies, including Thomas’ equation, in our population, then to compare these performances with those obtained by similar studies conducted in the context of developed countries. Therefore, we limited ourselves to examining whether, conceptually, this prediction of pre-pregnancy weight based on the characteristics of the woman considered in Thomas’ equation could pave the way for adopting such an approach, like what is done with the IOM references for gestational weight gain, for example.

3. Pre-pregnancy weight is usually relevant for the calculation of pre-pregnancy BMI. Several studies in the field have looked at how using different weights (self-reported, measured pre-pregnancy, first trimester, etc.) would impact pre-pregnancy BMI classification. This is usually done by calculating the Kappa coefficient. This is a relevant analysis that needs to be included

Thanks for the useful suggestion. Kappa coefficients analysis is now included in the method section (line 193 -196), in the results (lines 325-328 and Table 2 at page 16) and in the discussion (lines 444-445).

4. Do the authors have collected self-reported pre-pregnancy weight? If so, why not evaluate the agreement of that with the gold standard as well?

Unfortunately, self-reported pre-pregnancy weight was not collected. Indeed, the secondary analysis that we present in our manuscript was not foreseen when the study was set up. We have included this point in the limitations section (lines 460-462).

5. It is not correct to use Pearson’s correlation coefficient to evaluate agreement between two variables. Please refer to 10.1016/j.theriogenology.2010.01.003 for more information.

Thanks for sharing that interesting and useful paper. The Pearson’s correlation coefficient components have been removed from the methods (line 235) and the results (line 314 and Table 2 at page 16). Consequently, we also suppressed any reference to Pearson’s correlation coefficient when we compared our results to those from other studies (lines 397 to 400 and lines 415-417). Please note that for more clarity we have also added the word “concordance” before “correlation coefficients” since we still use the Lin correlation coefficients for gauging the agreement (e.g Table 2 at page 16 and lines 374, 377, 426, 434).

6. How do the 302 women selected for this study compare to the 411 women who became pregnant in the RECIPAL cohort?

Thanks for the comment. A table comparing the 302 selected women to the 109 unselected women of RECIPAL study was added as a supplementary table (please see S1 Table in the supporting information file and lines 297-299 in the results section). The women who were not included in the analysis tended to be taller and larger in body size. They were more often from the first site of the study (So-Ava) than from the second one (Akassato) and were more frequently of the Toffin ethnic group. The RECIPAL study was initially planned to be conducted exclusively within the lake-dwelling community of So-Ava. A sample of 500 pregnant women was targeted but it happened to be difficult to reach this goal within the sole So-Ava community for several reasons, including cases of refusal, among others. It was therefore decided to extend the study area to a neighboring locality (Akassato). The women from So-Ava, who are primarily of the Toffin ethnicity, were therefore followed for a longer period during the pre-pregnancy period (since the study began with them), and for some of them this led their latest pre-pregnancy weight measurement to be more than three months old when they became pregnant because weights were no longer measured after one year of pre-pregnancy follow-up. There were also more cases of withdrawal of consent among the Toffin women, who also happened to be larger in body size. These differences were therefore only due to a different study timing between the two locations and there is no obvious reason to think that this could have introduced a bias in our analysis. A shortened version of the above explanation has been added in the limitations section of the discussion (lines 462 to 469).

7. The end of the statistical analysis section is not clear.

We have entirely reworded the statistical analysis section with a special focus on the last paragraph to make it clearer, as suggested by the reviewer. This gave us the opportunity to enhance readability throughout the statistical analyses section (lines 175 to 216).
