# Supplementary material for: Comparison of two proxies for the preconception weight using data from a pre-pregnancy cohort in Benin: Weight measured in the first trimester of pregnancy vs estimated by Thomas’ formula
Source: PLoS One. 2024 Nov 4;19(11):e0312840. doi: 10.1371/journal.pone.0312840 (PMC11534216; doi:10.1371/journal.pone.0312840)
Supplement: Attachment — Submitted filename: Response to reviewers_Oct11.docx [file pone.0312840.s008.docx]

**Dear editor,**

We sincerely appreciate the opportunity to resubmit our revised manuscript, entitled “Comparison of two proxies for the preconception weight using data from a pre-pregnancy cohort in Benin: weight measured in the first trimester of pregnancy vs estimated by Thomas’ formula”.

We have carefully considered all the journal requirements and your additional comments to further strengthen the quality of our work.

Below, we provide detailed responses to each of the suggestions and explain the specific changes made to the manuscript.

We trust that these revisions address the remaining concerns and enhance the clarity and robustness of our study.

With kind regards,

Dr Emmanuel Koffi YOVO

Clinical Research Institute of Benin (IRCB)

04 BP 1114, Abomey-Calavi

Email: emkoffiyovo@gmail.com

Tel: +229 97 42 08 01

**Response to the academic editor**

**Journal Requirements:**

Please review your reference list to ensure that it is complete and correct. If you have cited papers that have been retracted, please include the rationale for doing so in the manuscript text or remove these references and replace them with relevant current references. Any changes to the reference list should be mentioned in the rebuttal letter that accompanies your revised manuscript. If you need to cite a retracted article, indicate the article’s retracted status in the References list and also include a citation and full reference for the retraction notice.

We reviewed all references, the context, and the rationale for their inclusion in the article, starting from the original submitted version to the first revised version. The clarifications are as follows:

1. In the initial version of the submitted manuscript, reference number 20 was automatically re-identified by Zotero reference management software as reference 33. During the first revision process, this error was corrected, and reference number 20 was uniquely assigned to this article, which has been consistently maintained in the paper.

2. Additionally, during the revision process, reference 27, which was cited in the Methods section of the initial submitted version, provided details on the restricted cubic splines method used for the analysis. Given that this method is already well-known, the reference was ultimately deemed unnecessary and was removed from the revised version.

3. In accordance with the reviewer 2 suggestion regarding the Kappa coefficient, we have added reference 29 to justify the inclusion of Kappa coefficient analyses in the revised version of the manuscript.

We have made a concerted effort to thoroughly address the reviewers' comments, but we inadvertently omitted to clarify these changes regarding references, which were made on our own initiative. Thank you for giving us the opportunity to clarify this.

**Additional Editor Comments:**

1) The reviewer’s comments have been adequately addressed. Minor comments remain with respect to presentation.

Thanks.

We have taken all the suggestions into account, and the lines indicated below allow for verification in the tracked changes version of the manuscript.

2) Delete ‘rather’ wherever it occurs in the text. It is unscientific.

“Rather” deleted on lines: 45; 80; 221; 290 and 316.

3) There is no need to include the STATA code.

STATA codes are removed from the manuscript on lines: 158; 164; 167; 175-176 and 180.

All the following suggestions have been considered:

198: delete sort of (Done, line 170)

200 delete thus (Done, line 172)

212: delete as above (Done, line 184)

216: delete also (Done, line 188)

290: delete the “” (Done, line 207)

297: don’t capitalize Sociodemographic (Done, line 212)

453: delete In particular, thanks to (Done, line 355)

462: delete for example (Done, line 364)
